# A Unique Publication Model that Works

**DOI:** 10.5041/RMMJ.10323

**Published:** 2018-01-29

**Authors:** Shraga Blazer

**Affiliations:** Editor-in-Chief, Rambam Maimonides Medical Journal

Dear Friends and Colleagues,

*Rambam Maimonides Medical Journal* was once a new and unknown publication. Today we have more than 17,000 subscribers from 146 nations and territories. We published 39 scientific medical papers in 2017 out of 61 submitted manuscripts.

We are now indexed by PubMed and Thompson Reuters Emerging Sources Citation Index, to name a few. Next year, the Journal is scheduled to receive an official impact factor from Thompson Reuters.

We are not so unknown anymore.

As a new Journal, most of the papers submitted were naturally reviews. However, the most important aspect for the promotion and advancement of medicine is publication of original research. To promote such efforts the editors of *Rambam Maimonides Medical Journal* established in 2017 the Maimonides Best Published Original Research Prize. This annual prize of $1,000 is to be awarded to the first author of the best original research paper published in the journal over the previous year.

This prize is an added benefit to authors. Since asking for fees from authors who have few or no research funds or grants may preclude publication of their papers, our Journal has no subscription fees, no submission fees, and no publication fees. Equally noteworthy is that although published by Rambam Health Care Campus, the Journal is unaffiliated with any organization or society. A decision was also made when the Journal was established to seek *no* advertising—perhaps the most unusual feature of this publication. *Rambam Maimonides Medical Journal* is donor-supported by those who share in our vision to ensure that anyone anywhere in the world can submit their scholarly work with no fear of commercial bias and no fees to impede publication.

This editorial effort has been fruitful and has led to excellent results. As we enter our ninth year of publication, it is rewarding to note that *Rambam Maimonides Medical Journal* has brought to the world 273 original scientific medical papers on a variety of topics.

With the announcement of the Maimonides Best Published Original Research Prize, 25% of our published papers in 2017 were original research articles. Determining the best published paper from so many well written articles presenting many important research findings was no small task. Nevertheless, after careful deliberation, the RMMJ Original Research Prize Committee selected a winner.

I am pleased to announce that the first recipient of this prize is Dr. Anatoli Stav for his paper titled “Femoral versus Multiple Nerve Blocks for Analgesia after Total Knee Arthroplasty,”^1^ with co-authors Leonid Reytman, Roger Sevi, Michael Yohay Stav, Devorah Powell, Yanai Dor, Mickey Dudkiewicz, Fuaz Bayadse, Ahud Sternberg, and Michael Soudry.

As a general medicine publication, *Rambam Maimonides Medical Journal* has the freedom to present topics of varied and specific interest. To that end, we not only ensure publication of papers with a Jewish flavor,^e.g.^^2^–^4^ we also include important though unusual topics, such as determining diagnoses through art^e.g.^^5^,^6^ and history.^e.g.^^7^ In addition, we dedicate at least one to two issues per year on a special topic of interest worldwide, with quite positive feedback from our readership.

The January 2018 issue of *Rambam Maimonides Medical Journal* includes a special supplement: Abstracts from the 14th Annual Rambam Research Day. We would like to encourage other medical organizations to contact us regarding publication of supplements representing the research from their esteemed institutes.

Our hope is that *Rambam Maimonides Medical Journal* will serve the international scientific medical community with a broad range of scholarly papers that might not have been published via any other venue.

## References

[1] Stav A, Reytman L, Sevi R (2017). Femoral versus multiple nerve blocks for analgesia after total knee arthroplasty. Rambam Maimonides Med J.

[2] Friedman AN (2016). Medical ethics in nephrology: a Jewish perspective. Rambam Maimonides Med J.

[3] Greenberger C, Mor P (2016). Should Sabbath prohibitions be overridden to provide emotional support to a sick relative?. Rambam Maimonides Med J.

[4] Urkin J, Fram E, Jotkowitz A, Naimer S (2017). Nurturing a society of learners: suggestions from traditional Jewish pedagogy for medical education. Rambam Maimonides Med J.

[5] Weisz GM, Albury WR (2015). Diseases of old age in two paintings by Rembrandt. Rambam Maimonides Med J.

[6] Lazzeri D, Lippi D, Castello MF, Weisz GM (2016). Breast mass in a Rubens painting. Rambam Maimonides Med J.

[7] Duek I, Cohen JT, Gil Z (2018). Unilateral vocal cord paralysis of a great Jewish opera singer. Rambam Maimonides Med J.

